# Comparison of Pain Management Strategies to Reduce Opioid Use Postoperatively in Free Flap Breast Reconstruction: Pain Catheter versus Nerve Block in Addition to Refinements in the Oral Pain Management Regime

**DOI:** 10.1055/s-0043-1777673

**Published:** 2024-02-29

**Authors:** Andrea B. Stefansdottir, Luis Vieira, Arni Johnsen, Daniel Isacson, Andres Rodriguez, Maria Mani

**Affiliations:** 1 Department of Plastic and Reconstructive Surgery, Uppsala University Hospital, Uppsala, Sweden; 2 Department of Surgical Sciences, Uppsala University, Uppsala, Sweden; 3 Department of Plastic Surgery, Central University Hospital Center, Lisbon, Portugal; 4 Department of Otorhinolaryngology, Landspitali, National University Hospital of Iceland, Reykjavik, Iceland

**Keywords:** free flap breast reconstruction, pain management, DIEP

## Abstract

**Background** Perioperative management in autologous breast reconstruction has gained focus in recent years. This study compares two pain management protocols in patients undergoing abdominal-based free flap breast reconstruction: a past protocol (PP) and a current protocol (CP)—both intended to reduce opioid consumption postoperatively. The PP entails use of a pain catheter in the abdominal wound and the CP consists of an intraoperative nerve block in addition to refinements in the oral pain management. We hypothesize that the CP reduces opioid consumption compared to PP.

**Methods** From December 2017 to January 2020, 102 patients underwent breast reconstruction with an abdominal-based free flap. Two postoperative pain management strategies were used during the period; from December 2017 to September 2018, the PP was used which entailed the use of a pain catheter with ropivacaine applied in the abdominal wound with continuous distribution postoperatively in addition to paracetamol orally and oxycodone orally pro re nata (PRN). From October 2018 to January 2020, the CP was used. This protocol included a combination of intraoperative subfascial nerve block and a postoperative oral pain management regime that consisted of paracetamol, celecoxib, and gabapentin as well as oxycodone PRN.

**Results** The CP group ( *n* = 63) had lower opioid consumption compared to the PP group ( *n* = 39) when examining all aspects of opioid consumption, including daily opioid usage in morphine milligram equivalents and total opioid usage during the stay ( *p* < 0.001). The CP group had shorter length of hospital stay (LOS).

**Conclusion** Introduction of the CP reduced opioid use and LOS was shorter.

## Introduction

Abdominal-based free flap breast reconstruction is considered gold standard in breast reconstruction. 1 Despite its increasing popularity and widespread application worldwide, it is still considered a major engagement and task for both patients and the health care system in many institutions. Improvement in perioperative care may lead to increased availability of the procedure to a larger population, at lower risks and costs. Thus, it is important to find ways to optimize the different steps of abdominal-based free flap breast reconstruction, and optimizing postoperative pain management is one such step.

Introduction of enhanced recovery after surgery (ERAS) pathways has shown to decrease stress response after surgery and improve surgical outcome in noncardiac surgical patients. 2 Optimal pain management is a part of the ERAS concept. Previous studies have shown that appropriate pain control postoperatively motivates early mobilization and is associated with less complications and shorter hospital stays. 3 4 New multimodal pain control protocols and modified surgical techniques in abdominal-based breast reconstruction have attempted to improve pain control. 5 6 7 8 9 10 11

The aim of the current study was to compare two different postoperative pain management protocols that both were designed to reduce postoperative opioid usage in patients undergoing abdominal-based free flap breast reconstruction; a past protocol (PP) and a current protocol (CP) as described below. The hypothesis is that the CP generates lower use of opioids postoperatively (primary outcome) and is associated with less side effects related to the pain management compared to the PP. CP is also thought to contribute to a shorter length of hospital stay (LOS; secondary outcomes) compared to the PP.

## Methods

### Study Design

All patients who underwent abdominal-based free flap breast reconstruction at Uppsala University Hospital, Sweden, from December 2017 to January 2020, were included in the study. The standard operation method used was the deep inferior epigastric perforator (DIEP) flap with only a few exceptions were the superficial inferior epigastric artery flap or the muscle-sparing transverse rectus abdominis myocutaneous flap was used. The study was approved by the Regional Ethical Committee (Dnr 2014/354).

Data were retrieved retrospectively from the hospitals' electronic patient records, including age at surgery, weight, body mass index (BMI), comorbidities, previous medical history, intraoperative details, postoperative pain medication consumption, postoperative complications, side effects from opioid consumption and reoperations. Patient's previous medical history was examined with respect to comorbidities and history of a previous pain diagnosis was documented. Intraoperative details included type of flap used, whether unilateral or bilateral reconstruction, site of anastomosis, surgical method used for exposing recipient vessels (rib-sparing or rib-sacrificing) as well as method of abdominal closure. LOS was defined as the number of days from the day of operation until discharge. The follow-up period was 30 days postoperatively.

**Fig. 1:**
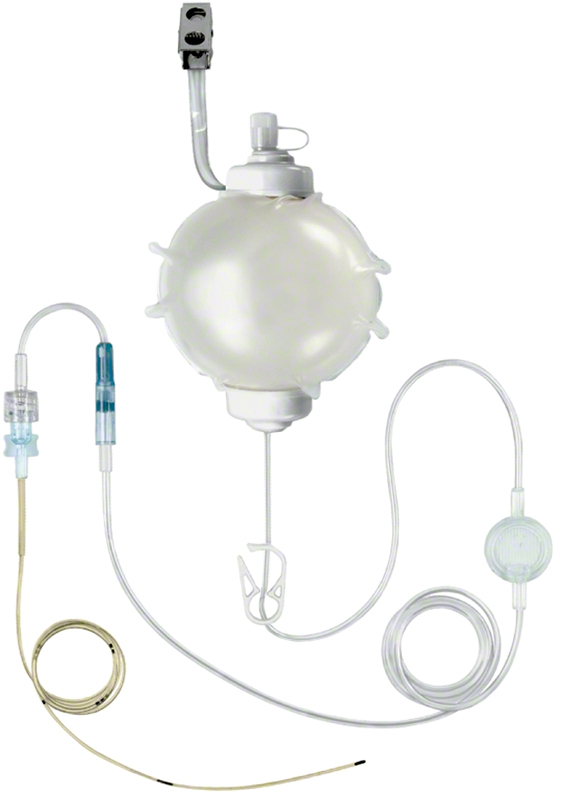
Pain pump or pain buster, the cornerstone of pain management in PP group. PP, past protocol.

During the study period, two different postoperative pain management protocols were used, the PP was used from December 2017 to September 2018. The cornerstone of the pain management in the PP group was pain catheters that were connected to pumps (so-called pain busters), in addition to oral pain management with paracetamol (1,000 milligram [mg]) every 6 hours and fast-acting opioids as needed for breakthrough pain orally (oxycodone 5 mg). The patients in the PP group each had two pain catheters, one on each side of the abdomen placed intraoperatively. The pain catheters were placed under the rectus fascia and connected to a pain pump. The catheters were left in place and sutured or taped to the skin after abdominal closure. Each pain pump was filled with 100 milliliters (mL) of ropivacaine, 2 mg/mL, that was continuously dispersing ropivacaine at the donor site at approximately 2 mL/hour. Fig. 1 shows the pain catheter/pump used. The pain catheters/pumps were monitored every 6 hours and removed on doctors' orders, as a standard, on postoperative day 2.

From October 2018 until January 2020, the CP was followed. The CP included intraoperative subfascial nerve block at the abdominal site. The nerve block was administered before closure of the abdominal wound. Two syringes filled with 20 mL of ropivacaine 2 mg/mL each were used to inject the analgesia under the exposed anterior layer of the rectus sheath, 20 mL of ropivacaine was administered on each side of the abdomen in a random fashion. No pain catheters/pumps were used. The oral pain management for the CP group consisted of paracetamol (1,000 mg) every 6 hours, nonsteroidal anti-inflammatory COX-2 inhibitor every 12 hours (celecoxib 200 mg) and gabapentin (300 mg) every 8 hours and fast-acting opioids as needed for breakthrough pain orally (oxycodone 5 mg).

The aim for all patients was to keep postoperative pain at an acceptable level with a visual analogue scale (VAS) pain score below 3. 12 Nurses on the ward monitored the pain scores and administered oxycodone pro re nata (PRN) for pain relief to achieve VAS less than 3. All patients stayed 1 to 4 hours postoperatively in the postoperative recovery unit and were transferred to the plastic surgery ward thereafter. The two different pain management protocols are listed in Table 1 .

**Table 1:** Pain management in the past protocol and the current protocol groups

	PP group	CP group
Scheduled or around the clock	Ropivacaine 2 mg/mL; rate 2 mL/hour continuous infusion; volume: 100 mL on each side of abdomen; duration: 2 days	Ropivacaine 2 mg/mL; once; volume 20 mL on each side of abdomen; duration: single bolus
	Paracetamol (acetaminophen) 1,000 mg four times/day	Paracetamol (acetaminophen) 1,000 mg four times a day Gabapentin 300 mg thrice a day Celecoxib 200 mg twice a day
As needed (PRN)	Oxycodone 5 mg PRN	Oxycodone 5 mg PRN

Abbreviations: CP, current protocol; mg, milligram; mL, milliliter; mg/mL, milligrams per milliliter; PP, past protocol; PRN, pro re nata.

### Outcome Measures

Information regarding patient's opioid usage was retrieved from the patient's electronic notes. All opioids given were converted to morphine milligram equivalents (MMEs). 13 As a standard, fast-acting opioid (oxycodone 5 mg) tablets were used for breakthrough pains.

### Data Analysis

All data were collected jointly by two authors (A.B.S. and L.V.). Data were analyzed using R version 4.0.5. Continuous variables are reported as mean and standard deviation if normally distributed or as median and interquartile range, if not. Normality of data distribution was assessed by inspection of histograms and Q–Q plots. Categorial variables are presented as numbers of patients and percentages. For the normally distributed continuous variables, a *t* -test was used and Mann–Whitney U test was used for non-normally distributed continuous variables. A chi-square test was used for group variables, except in the cases with a low expected count (<5) where a Fisher's exact test was used. When comparing the opioid usage between the groups, a Mann–Whitney U test was used both when comparing total use of opioids in MME, day-specific opioid use in MME and when comparing the total use in MME per kilogram (kg), as these variables were non-normally distributed. A multivariate linear regression was used to assess whether BMI, previous pain diagnosis, previous comorbidities, or intraoperative variables (rib sacrifice) which differed between the groups were risk factors for excess opioid usage. A *p* -value of <0.05 was considered statistically significant.

## Results

One hundred and two patients underwent breast reconstructive surgery with an abdominal-based free flap during the study period ( Table 2 ). Of the 102 patients included in the study, 39 were treated according to the PP and 63 patients were treated according to the CP. There were neither differences between groups as for age, weight, BMI, previous radiation to the chest wall nor previous pain diagnosis ( *p* > 0.05; Table 2 ).

**Table 2:** Baseline characteristics of the past protocol group and the current protocol group

Characteristics	Patient group		*p* -Value
	PP, *n* = 39	CP, *n* = 63	
Age at surgery, y	52.0 ± 9.8	51.1 ± 9.4	0.67
Weight, kilograms	73.6 ± 12.3	72.6 ± 9.4	0.66
Body mass index, kg/m ^2^	26.2 ± 3.0	26.3 ± 2.9	0.88
Previous radiation	26 (66.7)	45 (71.4)	0.78
Previous pain diagnosis	8 (20.5)	11 (17.5)	0.90
Any comorbidity	30 (76.9)	45 (71.4)	0.70

Abbreviations: CP, current protocol; PP, past protocol; y, years.

Values are listed as mean ± standard deviation or number of patients (%). Level of significance was set at *p* < 0.05.

### Intraoperative Data

The PP and the CP groups were very similar with no statistically significant difference shown between the groups for type of reconstruction (immediate or delayed), flap used, type of abdominal closure method, or the rate of symmetrizing contralateral surgery (breast reduction or mastopexy). The DIEP flap was chosen for 92.3% of the patients in the PP group and 89% of the patients in the CP group ( *p* = 0.84). In both groups, a unilateral reconstruction was more common than bilateral, 87.2% in the PP group and 76.2% in the CP group ( *p* = 0.27). In both the groups, a traditional abdominal closure with sutures was used for majority of patients with no need for a fascia plication or mesh, 94.9% received a traditional closure with sutures in the PP group and 90.5% in CP group ( *p* = 0.67). There were no differences between the groups for any of the intraoperative characteristics researched, except regarding rib technique used for exposure of receiving vessels. In the PP group, rib-sacrificing technique was more commonly used, or for 46.2% of the patients compared to 23.8% in the CP group ( *p* = 0.03). Multivariate analysis was done because of this difference between the groups that showed that rib sacrifice did not correlate with a higher opioid consumption ( *p* = 0.44) and rib sacrifice was, therefore, not considered a confounding factor in this study.

### Postoperative Outcomes

**Fig. 2:**
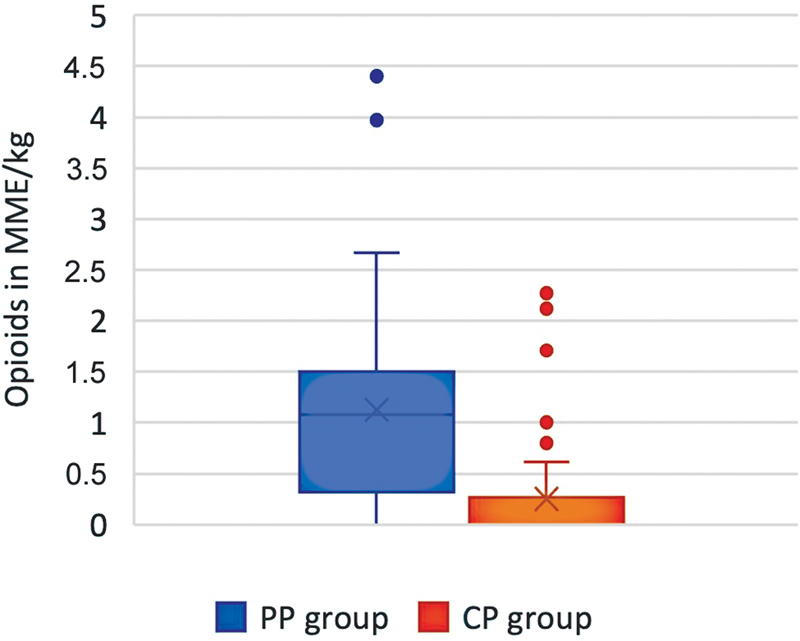
Comparison of total opioid consumption in morphine milligram equivalents per kilogram body weight during hospital stay. CP, current protocol; MME, morphine milligram equivalent; PP, past protocol.

The hypothesis was that CP reduced opioid consumption when compared to the PP. The results showed that the CP group had lower opioid consumption in all aspects. The CP group used less opioids compared to the PP group for postoperative day (POD) 1 (POD1) to POD4, *p* < 0.001 ( Table 3 ). When analyzed, the total opioid consumption during the hospital stay, in MME per kilogram body weight, was lower in the CP group with a median value of 0 mg/kg (0.0, 0.3) compared to 1.1 mg/kg (0.4, 1.4) in the PP group ( *p* < 0.001; Fig. 2 ). A separate comparison of total MME of opioids during the stay showed a median of 0 MME (0, 20) in the CP group compared to a median of 60 MME (30, 112) in the PP group ( *p* < 0.001). Opioid consumption decreased from POD1 to POD4 in both groups ( Table 3 ).

**Table 3:** Comparison of opioid consumption in morphine milligram equivalent per day between groups on postoperative days 1 to 4

Opioid consumption in MME, median (IQR)	Patient group		*p* -Value
	PP group	CP group	
Opioids on POD1	20 (10, 30)	0 (0, 10)	<0.001
Opioids on POD2	20 (10, 40)	0 (0, 0)	<0.001
Opioids on POD3	10 (0, 20)	0 (0, 0)	<0.001
Opioids on POD4	0 (0, 20)	0 (0, 0)	<0.001
Total opioid consumption during hospital stay in MME, median (IQR)	60 (30, 112)	0 (0, 20)	<0.001
Length of hospital stay in days, median (IQR)	5 (5, 5)	4 (4, 5)	<0.001

Abbreviations: CP, current protocol; IQR, interquartile range; MME, morphine milligram equivalent; POD, postoperative day; PP, past protocol.

All opioid variables are non-normally distributed. Groups are compared with nonparametric tests. Values are listed as median opioid consumption in milligrams (IQR). *p* -Value was set at <0.05.

Linear regression analysis of BMI, previous pain diagnosis and comorbidities could not identify any relation of these factors to increased total opioid use, for neither of the groups (PP or CP).

The LOS was shorter for the CP group with a median stay of 4 days (4, 5) compared to 5 days (5, 5) in the PP group ( *p* < 0.001).

### Complications

There was no difference in 30-day complication rates between the groups with the overall complication rate in the CP group being 27.0% compared to 35.9% in the PP group ( *p* = 0.47). Reoperation rates in the CP group were 9.5% compared to 5.1% in PP group (0.71). Reasons for reoperations were vein thrombosis ( *n* = 2), arterial thrombosis ( *n* = 3), fat necrosis ( *n* = 1), mastectomy flap necrosis ( *n* = 2), and hematoma ( *n* = 2). One of the patients in the PP group had two takebacks to surgery, the first one due to a hematoma and an arterial thrombosis and the second due to venous thrombosis. Postoperative infections were found similar for both patient groups, 12.7% in the CP group and 10.3% in the PP group ( *p* = 1.0). There was one total flap failure (1%) which was in the PP group.

There were no serious complications resulting in reoperations or permanently damaging incidents related to either of the local pain management protocols (neither pain catheter/pump in PP group nor subfascial nerve block in CP group). Potentially opioid-related complications such as nausea and headache were similar between the groups, see Table 4 .

**Table 4:** Potential opioid-related events

Event	Patient group		*p* -Value
	PP group, *n* = 39	CP group, *n* = 63	
Nausea, *n* (%)	10 (25.6)	18 (29)	0.95
Headache, *n* (%)	1 (2.6)	4 (6.5)	0.71

Abbreviations: CP, current protocol; n, number of patients; PP, past protocol.

Values are listed as number of patients ( *n* ) and percentages (%). *p* -Value was set at <0.05.

Information on first bowel movement postoperatively was missing for a large part of the patient population and that is why no further analysis was performed.

## Discussion

Breast reconstruction with abdominal-based free flap is a potentially complex procedure. Over the years, the intraoperative technical refinements have been improved and subsequently, the perioperative management has gained focus in order to optimize care and resource management. 8 9 Pain management protocols aim to achieve successful baseline pain control, avoid breakthrough pain episodes, associated rescue painkiller usage and their side effects. The current study compares two different pain management protocols and found that patients treated with the PP protocol received more than three times as much opioids compared to the CP group on POD1 and more than four times as much on POD2. These pain management protocols differ in more than one way and that is considered one of the limitations of this study. The patients following the PP received local anesthesia via pain catheter/pump and their oral regime consists of scheduled paracetamol and oxycodone PRN. The patients following the CP receive an intraoperative nerve block instead of the pain catheters/pump and the oral regime was not only scheduled paracetamol and oxycodone PRN but they also received scheduled gabapentin and celecoxib orally. We can assume that the combination of the analgesia used in the CP group is associated with less consumption of opioids postoperatively, although we cannot be certain which treatment parameter is the most important.

Vasoconstriction is a potential cause of flap failure and may be caused by pain as well as hypovolemia and hypothermia. As a consequence, adequate postoperative pain management in free flap surgery is crucial. 14 15 The aim for the patients of the current study was to keep the patient reported VAS 12 pain score at 3 or less. The current study had one flap failure (total flap failure rate of 1.0%), which is coherent to previous reports, 16 suggesting that the patients did not suffer from an unreasonable amount of pain resulting in unexpected high level of flap failure in either of the pain protocol groups.

Despite a remarkably higher usage of opioids in the PP group, there was no difference in reported possible opioid-related side effects such as headache, nausea, or vomiting. This may be due to inadequate registration in the hospital records when patients report minor symptoms of nausea or headache. We speculate that another possibility for the indifference between the groups is that the patients in the CP group might have had gabapentin-related side effects similar to the opioids' side effects (headache and nausea), however, no further specific analysis was possible within the frame of the current study.

Gabapentin, in previous studies, has been shown to be related to altered mental status, primarily in the elderly population; 17 we found no such correlations in our population. Notably, the patients in the current study only received gabapentin for a short period of time, only while hospitalized postoperatively. Both gabapentin and celecoxib have opioid-sparing effects, large meta-analyses have associated gabapentin with decreasing opioid usage and lower VAS pain scores postoperatively. 18 The combination of these drugs has shown benefits in previous studies, as in this study. 19

Kulkarni et al demonstrated that patients undergoing bilateral reconstruction perceived more severe postoperative pain as well as evidence suggesting that patients undergoing immediate breast reconstruction having more severe discomfort and pain suffering. 20 In the current study, the two groups had comparable percentage of patients undergoing immediate reconstruction as well as bilateral reconstruction. As such these factors are not likely to explain the difference in opioid use between the groups. The percentage of patients who underwent a simultaneous symmetrizing surgery with a mastopexy or a breast reduction was similar between the groups. It remains unclear if these patients perceive more pain than the patients undergoing unilateral free flap breast reconstruction with no contralateral surgery, but previous studies have suggested that a symmetrizing surgery at the same time as a breast reconstruction with a free flap can be beneficial to the patients. 21

The technique used for internal mammary vessel exposure may affect postoperative pain. When using the rib-sparing technique, the internal mammary vessels are approached and exposed in the rib interspace rather than removing rib cartilage. Rib sacrifice is considered more painful than rib sparing, 22 and previous studies have shown that rib-sacrificing techniques correlate with more opioid consumption postoperatively in patients undergoing DIEP flap breast reconstruction. 6 23 In the current study, the rib-sacrificing technique was used to a larger extent in the PP group, however, multivariate analysis did not confirm rib technique as a confounding factor.

The CP group had shorter LOS than the PP group with a median LOS of 4 days compared to 5 days in the PP group. It is worth noting that throughout the study period there was a continuous effort to encourage early discharge which makes it difficult to state that the observed shortened LOS is solely due to the changes in pain management. The shorter LOS seen in the CP group is likely due to the trend seen in recent years towards shorter LOS for DIEP patients. The LOS has, to some degree, been standardized and aimed at 4 days for patients undergoing unilateral DIEP breast reconstruction at Uppsala University Hospital. Previous studies have shown that fast-track recovery pathways such as the ERAS pathway can shorten the LOS, 24 of which the pain management is one aspect. Of note, the decrease in LOS in the current study did not correlate with an increase in complications. The incidence of complications was similar between both groups and comparable to results previously shown in the literature. 25

The CP has the advantage for the patient of two less invasive lines attached to the abdomen which might affect the patients' comfort and ability to mobilize. Our clinical experience coheres with this, and we find that removal of pain catheters/pumps promotes patient mobilization. Although we did not analyze costs in this specific study, the estimated cost of PP and its pain catheter/pump use is higher than applying an intraoperative nerve block as is done for the CP group. Other studies have not been able to determine the cost-effectiveness of pain catheters/pumps. 26 Most likely, a shorter LOS would be cost-effective, however, specific future studies need to analyze the cost-effectiveness of the current pain management protocols.

### Study Limitations

This is a single-center, retrospective study with a limited study population of 102 patients. While not randomized the patients had no baseline differences and no intraoperative differences, except regarding rib technique. As mentioned above the pain management differs between the groups in more than one way. The CP group has a more extended oral pain management regime, scheduled celecoxib and gabapentin, in addition to scheduled paracetamol and oxycodone PRN and the intraoperative nerve block whereas the PP group has the pain catheter/pump, scheduled paracetamol and oxycodone PRN but no celecoxib and gabapentin. Therefore, it is difficult to know which factor plays the biggest role in the refinements seen for the CP group. The aim was to keep the VAS score below 3 in both the PP and the CP groups but unfortunately VAS score was not documented precisely enough to be compared between the groups.

### Conclusion

The introduction of the described CP reduced the need for opioids postoperatively. Additionally, the CP group had a shorter LOS. In conclusion, the CP shows benefits compared to PP in abdominal-based free flap breast reconstruction.
